# Evaluating the Significance of Implementing Experience-Based Clinical and Public Health Training in Medical Education Systems to Overcome the Curricular-Practice Gap in Tertiary Healthcare Settings

**DOI:** 10.7759/cureus.95997

**Published:** 2025-11-03

**Authors:** Warda Raja, Aiza Bhatti, Kainat Raja, Babar Hayat, Bilal Masood, Zahid Azam Chaudry

**Affiliations:** 1 Pediatrics and Child Health, Agha Khan University Hospital, Karachi, PAK; 2 Internal Medicine, Al Nafees Medical College &amp; Hospital, ISRA University, Islamabad, PAK; 3 Dermatology, Al Nafees Medical College &amp; Hospital, ISRA University, Islamabad, PAK; 4 Health and Population, District Health Authority Attock, Attock, PAK; 5 Community Medicine, Bolan Medical College, Quetta, PAK; 6 Community Medicine, Nawaz Sharif Medical College, Gujrat, PAK

**Keywords:** attitude of health personnel, attitudes, community health services, health knowledge, medical education, practice, public health

## Abstract

Background

Clinical skills are often prioritized in medical education; however, it is essential to incorporate public health training as trainees' knowledge and practices vary according to their training and experience. The study was designed to evaluate the significance of implementing experience-based clinical and public health training in medical education systems to overcome the curricular-practice gap in tertiary healthcare settings.

Methods

A cross-sectional observational study was conducted between April 2025 and July 2025 involving 250 trainees, mainly final-year students (n=100), interns (n=80), and residents (n=70). A structured, self-administered Knowledge, Attitudes, and Practices (KAP) questionnaire was used to assess public health knowledge, attitude towards the integration into clinical practice, and self-report practices. Demographics and prior exposure to public health or health campaigns were also recorded. Analysis of variance (ANOVA) and Chi-square tests were used to compare KAP scores, while Pearson correlation was used to test the relationship between KAP domains, training level, and exposure.

Results

Interns and residents scored higher in terms of KAP than final year students (p<0.05). Adequate level of knowledge, positive attitudes, and active engagement were found in 162 (64.8%), 178 (71.2%), and 154 (61.6%) of the participants, respectively. The KAP domains showed strong positive correlations (r=0.580.66; p<0.001), and higher scores were substantially correlated with prior exposure to public health and campaign participation (p<0.05).

Conclusion

The degree of clinical training and exposure to public health had a significant impact on trainees' KAP. Integrating experience-based public health training into medical education systems can potentially overcome the curricular-practice gap in public health.

## Introduction

Public health knowledge is crucial for the improvement of outcomes of public health and linking the clinical care with preventive and community-based principles [1]. Although medical trainees tend to focus more on learning clinical skills, the inconsistent inclusion of public health concepts into their training may result in their inability to manage the wide determinants of health in clinical practice [2]. Clinical training and public health represent an ever-growing gap in medical training. This gap exists because public health is seen as a way to make physicians more holistic and community-focused in their approach to patient care, as well as to equip future doctors with skills in community-based health interventions [3].

Previous research has suggested that there is an uneven level of understanding of the principles of public health among medical trainees, and the attitudes toward implementing these principles in everyday clinical practice can be affected by the degree of clinical experience and previous exposure to public health activities [4]. Moreover, self-reported participation in preventive or community health activities is sometimes constrained even among those who possess sufficient theoretical knowledge [5]. These findings indicate that the attitudes of medical students, exposure to public health concepts during medical training, involvement in health campaigns, and systematic community-based learning could enhance their practical involvement in health promotion activities [6]. It has been reported that medical programs incorporating both experiential public health training and clinical rotations prepare the trainees for better learning and applying the concept of public health in treating patients [7]. Regardless of this fact, there is a lack of systematic evaluation of medical trainees' knowledge, attitudes, and practices (KAP), particularly when structured public health exposure varies [8].

The present study aimed to assess the KAP of the medical trainees in the field of public health and also to determine how the level of clinical training and previous exposure to the field of public health affected the KAP of the medical trainees [9]. This study also aimed to inform the approaches in building an effective bridge between clinical and public health training in medical education by identifying gaps and correlations between KAP scores.

## Materials and methods

This cross-sectional study was carried out in the clinical departments of multiple tertiary care teaching hospitals, mainly in collaboration with Nawaz Sharif Medical College, Al Nafees Medical College, and Bolan Medical College, all located in Pakistan, between April 2025 and July 2025. The study employed a consecutive non-probability sampling methodology. The Institutional Review Board of Al Nafees Medical College & Hospital granted an ethical approval waiver (Ref: 03/2025/MS/37) for the study, and informed consent was obtained from all participants.

A total of 250 trainees were recruited, including medical final-year students (n=100), interns (n=80), and residents (n=70). OpenEpi version 3.0.0 (Dean AG, Sullivan KM, Soe MM. OpenEpi: Open Source Epidemiologic Statistics for Public Health, Version. www.OpenEpi.com, updated 2013/04/06) was used to determine the sample size, 95% level of confidence, margin of error of 5%, and assumed prevalence of sufficient KAP scores (50%), yielding the minimum number needed of 218 participants [9]. The inclusion criteria included trainees currently enrolled in clinical programs who were willing to respond to the questionnaire. All those trainees who were absent during rotations, had incomplete responses, and those with prior experience in professional practitioner areas on various community health issues were excluded.

A self-administered structured observational questionnaire was developed after examining validated tools from the literature on medical education and approved by two local content experts [10,11]. The tool was created to evaluate three areas: (i) self-reported practices about preventive and community health initiatives; (ii) attitudes towards incorporating public health into clinical training; and (iii) knowledge of public health principles and their relevance to clinical practice. Additionally, demographic data was gathered, such as age, gender, training level, previous public health exposure, and involvement in health campaigns. Participants' responses were scored and divided into groups on the basis of their active participation in practices, positive attitudes, and sufficient knowledge. The Appendix contains the complete questionnaire.

IBM SPSS Statistics for Windows, Version 26 (Released 2019; IBM Corp., Armonk, New York, United States) was used to analyze the data. KAP scores and other continuous variables were expressed as mean ± SD and compared across training groups using analysis of variance (ANOVA). Chi-square tests were used to compare categorical variables, which included percentages of participants with adequate knowledge, positive attitudes, and active practices. These variables were expressed as n (%). The associations between KAP scores, training level, and previous exposure to public health were evaluated using Pearson correlation. The threshold for statistical significance was set at p<0.05. 

## Results

A total of 250 medical trainees participated in this study to assess their KAP regarding public health and its integration into clinical training. Individuals who had prior exposure to public health concepts or had involvement in health campaigns before demonstrated better knowledge, more positive attitudes, and greater engagement in community health-related clinical activities. Table 1 shows the demographic features of the population used in the study.

**Table 1:** Demographic characteristics of medical trainees (n=250) SD: Standard Deviation, n: number of participants, %: Percentage, *Significance at p<0.05, degree of freedom (df): 1

Parameter	Total (n=250), n (%)	Final-year students (n=100), n (%)	Interns (n=80), n (%)	Residents (n=70), n (%)	Statistical test	Test value	p-value
Age (years) (Mean ± SD)	24.8 ± 2.7	23.9 ± 2.5	24.6 ± 2.8	26.2 ± 2.6	ANOVA	10.21	<0.001*
Gender (Male)	132 (52.8%)	52 (52.0%)	44 (55.0%)	36 (51.4%)	Chi-square	0.29^df^	0.867
Gender (Female)	118 (47.2%)	48 (48.0%)	36 (45.0%)	34 (48.6%)			
Prior public health exposure	145 (58.0%)	48 (48.0%)	50 (62.5%)	47 (67.1%)	Chi-square	7.81	0.020*
Participation in health campaigns	98 (39.2%)	28 (28.0%)	36 (45.0%)	34 (48.6%)	Chi-square	9.64	0.008*

Most of the trainees were 23 to 27 years old, with residents being older on average. In contrast to final-year students, interns and residents had significantly higher levels of prior public health exposure and health campaign participation (p=0.020 and p=0.008, respectively), indicating more opportunities for integration during clinical training. Table 2 provides KAP scores of the medical trainees.

**Table 2:** Knowledge, Attitudes, and Practices (KAP) of medical trainees SD: Standard Deviation, n: Number of participants, %: Percentage, *Significance at p<0.05.

Parameter	Total (n=250)	Final-year students (n=100)	Interns (n=80)	Residents (n=70)	Statistical test	Test value	p-value
Knowledge (Mean ± SD)	18.4 ± 4.7	16.2 ± 4.1	19.3 ± 4.2	21.1 ± 4.5	ANOVA	22.68	<0.001*
Adequate knowledge, n (%)	162 (64.8%)	54 (54.0%)	55 (68.8%)	53 (75.7%)	Chi-square	11.92	0.003*
Attitude (Mean ± SD)	22.6 ± 5.1	20.1 ± 4.8	23.4 ± 5.0	25.3 ± 4.9	ANOVA	17.54	<0.001*
Positive attitude, n (%)	178 (71.2%)	60 (60.0%)	59 (73.8%)	59 (84.3%)	Chi-square	15.37	<0.001*
Practice (Mean ± SD)	19.8 ± 4.9	17.5 ± 4.5	20.2 ± 4.6	22.1 ± 5.1	ANOVA	14.87	<0.001*
Active engagement, n (%)	154 (61.6%)	54 (54.0%)	48 (60.0%)	52 (74.3%)	Chi-square	6.89	0.032*

The KAP scores of interns and residents were significantly higher than those of final-year students (p-value<0.05), suggesting that exposure to public health activities and structured clinical training facilitate the conversion of theoretical knowledge into clinical practice. Correlations between practice, attitude, and knowledge scores are shown in Table 3, along with associations with previous exposure and training year.

**Table 3:** Correlation between KAP scores, training level, and prior exposure Pearson r: correlation coefficient, KAP: Knowledge, Attitude, Practice, *Significance at p<0.05.

Variable pair	Correlation coefficient (r)	Statistical test	Test value	p-value
Knowledge vs. Attitude	0.63	Pearson’s r	12.4	<0.001*
Knowledge vs. Practice	0.58	Pearson’s r	11.2	<0.001*
Attitude vs. Practice	0.66	Pearson’s r	13.1	<0.001*
Year of training vs. KAP	0.49	Pearson’s r	8.5	<0.001*
Prior exposure vs. KAP	0.41	Pearson’s r	7.2	<0.001*

These correlations support the study's main goal of bridging the gap between public health and clinical training by indicating that knowledge strongly predicts both attitude and practice, and that clinical training level and prior exposure to public health greatly improve the integration of public health principles into clinical practice.

## Discussion

The current study assessed medical students' understanding, perspectives, and behaviors around public health and its relationship to clinical training. The results showed that most trainees had good attitudes (178 participants; 71.2%) and sufficient knowledge (162 participants; 64.8%), though active participation in public health activities was somewhat lower (154 participants; 61.6%). Compared to final-year students, interns and residents possessed noticeably higher KAP scores, suggesting that clinical experience improved comprehension and application of public health concepts. These findings demonstrated the significance of incorporating public health exposure into medical education in order to support comprehensive clinical practice.

KAP scores were found to be substantially positively correlated, indicating that trainees with a deeper understanding of public health were more likely to embrace positive attitudes and take an active part in community health and preventive initiatives [12]. Higher KAP scores were also linked to prior involvement in public health initiatives, such as health campaigns or community service, highlighting the value of experiential learning in connecting abstract ideas with real-world implementation [13]. These results were consistent with earlier studies showing that structured public health experiences enhance medical trainees' competency and engagement [14].

The study additionally demonstrated that KAP varied according to training level. Residents showed superior knowledge, attitudes, and practices due to their increased clinical exposure and responsibilities compared to interns and final-year students. This highlighted the importance of integrating public health principles longitudinally across various clinical training stages [15]. Accordingly, the findings support the idea that bridging occurred best when medical students were exposed to both structured clinical duties and real-world public health experiences simultaneously [16]. Preventive medicine workshops, community-based rotations, and public health modules could improve trainees' capacity to use public health principles in clinical decision-making [17,18]. 

The integration of public health and clinical training into a single, comprehensive approach to healthcare delivery could be achieved by improving public health education. This would better prepare future doctors to address social determinants of health, participate in community health programs, and apply preventive techniques in their clinical practice [19,20]. In addition to improving patient outcomes, bridging the gap between clinical training and public health fosters the development of a workforce capable of effectively addressing public health issues [21].

There are a number of limitations that must be acknowledged. As the data were self-reported, response bias might have been introduced. Additionally, the cross-sectional design makes it impossible to prove a causal relationship between training level and KAP outcomes. The study's generalizability was also limited as it was only carried out at some tertiary care hospitals from particular areas of the same country. Future research should incorporate multicenter longitudinal studies in order to determine whether early integration of public health experiences improved practice behaviors and patient outcomes, as well as the effect of structured public health curricula on clinical competencies over time.

## Conclusions

This study emphasizes the ways medical trainees exhibit varying public health KAP. Increased comprehension of public health principles leads to better participation in preventive and community health initiatives, as evidenced by the positive correlations found between knowledge, attitude, and practice. It can be further improved to overcome the curricular-practice gap in tertiary healthcare settings. These results were further enhanced by prior exposure to public health campaigns, illustrating the importance of experimental learning.

These findings highlight the way public health education needs to be systematically incorporated into clinical training to ensure that aspiring doctors are equipped to handle social determinants of health and narrow the gap between population health and individual patient care.

## Appendices

**Table 4:** Self-reported KAP questionnaire on bridging public health and clinical training among medical trainees This questionnaire was developed by the authors especially for this study. No outside sources were referenced, and no license or permission was needed. Scoring system: • Knowledge: 5 questions → Max 5 points (Adequate ≥ 3/5) • Attitudes: 5 items on 5-point Likert scale → Max 25 points (Positive ≥ 15/25) • Practices: 5 questions → Max 5 points (Active engagement ≥ 3/5) • Total KAP score: 35 points

Section A: Demographics			
Sr. No.		Questions	
1.		Age	______years
2.		Gender	☐ Male ☐ Female ☐ Other
3.		Year of Training	☐ Final-Year Student ☐ Intern ☐ Resident
4.		Prior Public Health Exposure	☐ Yes ☐ No
5.		Participation in Health Campaigns	☐ Yes ☐ No
Section B: Knowledge (Correct = 1, Incorrect = 0, Total = 5)			
6.	Public health focuses primarily on:		☐ Individual patient care ☐ Population health and prevention ☐ Hospital-based treatment ☐ Emergency care only
7.	Which is not a core function of public health?		☐ Health promotion ☐ Disease prevention ☐ Clinical surgery ☐ Policy development
8.	Social determinants of health include:		☐ Housing, education, income ☐ Only genetics ☐ Only hospital facilities ☐ None of the above
9.	Vaccination campaigns are an example of:		☐ Public health intervention ☐ Individual therapy ☐ Surgical care ☐ Emergency response
10.	Integration of public health into clinical practice can improve:		☐ Patient outcomes ☐ Preventive care ☐ Community health ☐ All of the above
Section C: Attitudes (5-point Likert scale: 1 = Strongly Disagree, 2 = Disagree, 3= Neutral, 4 = Agree, 5 = Strongly Agree, Total = 25)			
11.	Public health principles should be integrated into clinical training.		☐ 1 ☐ 2 ☐ 3 ☐ 4 ☐ 5
12.	Knowledge of social determinants of health is essential for medical trainees.		☐ 1 ☐ 2 ☐ 3 ☐ 4 ☐ 5
13.	Participation in community-based health activities enhances clinical skills.		☐ 1 ☐ 2 ☐ 3 ☐ 4 ☐ 5
14.	Preventive medicine is as important as curative medicine in clinical practice.		☐ 1 ☐ 2 ☐ 3 ☐ 4 ☐ 5
15.	I feel motivated to apply public health concepts in patient care.		☐ 1 ☐ 2 ☐ 3 ☐ 4 ☐ 5
Section D: Practices (Each response scored 0–1, Total = 5)			
16.	Have you ever participated in a vaccination or screening campaign?		☐ Yes (1) ☐ No (0)
17.	Do you routinely counsel patients about preventive health (diet, hygiene, lifestyle)?		☐ Yes (1) ☐ No (0)
18.	Have you worked in a community health program during training?		☐ Yes (1) ☐ No (0)
19.	Do you apply knowledge of social determinants in clinical decision-making?		☐ Always (1) ☐ Sometimes (0.5) ☐ Rarely (0) ☐ Never (0)
20.	In the past 6 months, how often have you engaged in public health–related activities?		☐ None (0) ☐ 1–2 times (0.5) ☐ 3–5 times (1) ☐ >5 times (1)

## References

[1] Zarei F, Dehghani A, Ratansiri A (2024). ChecKAP: a checklist for reporting a knowledge, attitude, and practice (KAP) study. Asian Pac J Cancer Prev.

[2] Kandasamy G, Almanasef M, Almeleebia T (2024). Knowledge, attitude and practice towards physical activity among healthcare students at a public university in Saudi Arabia. Front Public Health.

[3] Nour N, Onchonga D, Neville S, O'Donnell P, Abdalla ME (2024). Integrating the social determinants of health into graduate medical education training: a scoping review. BMC Med Educ.

[4] Punzalan JK, Guingona M, Punzalan MG, Cristobal F, Frahsa A, Liwanag HJ (2023). The integration of primary care and public health in medical students' training based on social accountability and community-engaged medical education. Int J Public Health.

[5] Church HR, Murdoch-Eaton D, Sandars J (2023). Under- and post-graduate training to manage the acutely unwell patient: a scoping review. BMC Med Educ.

[6] Kong Y, Zhu X, Yang Y, Xu H, Ma L, Zuo Y (2024). Knowledge, attitudes, practice, and public health education demand regarding PARI prevention: a cross-sectional study among Chinese undergraduates. Front Public Health.

[7] Peng Y, Pei C, Zheng Y, Wang J, Zhang K, Zheng Z, Zhu P (2020). A cross-sectional survey of knowledge, attitude and practice associated with COVID-19 among undergraduate students in China. BMC Public Health.

[8] Nemat A, Raufi N, Sediqi MF, Rasib AR, Asady A (2021). Knowledge, attitudes, and practices of medical students regarding COVID-19 in Afghanistan: a cross-sectional study. Risk Manag Healthc Policy.

[9] Bhardwaj R, Agrawal U, Vashist P, Manna S (2024). Determination of sample size for various study designs in medical research: a practical primer. J Family Med Prim Care.

[10] Yeazel MW, Lindstrom Bremer KM, Center BA (2006). A validated tool for gaining insight into clinicians' preventive medicine behaviors and beliefs: the preventive medicine attitudes and activities questionnaire (PMAAQ). Prev Med.

[11] Mukhopadhyay P, Dey I, Haldar A (2020). Development and validation of a tool to assess perceptions and practices regarding hypertension and associated comorbidities among primary health care providers of a rural community in India. Indian J Community Med.

[12] Xu H, Gonzalez Mendez MJ, Guo L (2020). Knowledge, awareness, and attitudes relating to the COVID-19 pandemic among different populations in central China: cross-sectional survey. J Med Internet Res.

[13] Ahmad Ghaus MG, Tuan Kamauzaman TH, Norhayati MN (2021). Knowledge, attitude, and practice of evidence-based medicine among emergency doctors in Kelantan, Malaysia. Int J Environ Res Public Health.

[14] Al-Qerem W, Eberhardt J, Jarab A (2023). Exploring knowledge, attitudes, and practices towards artificial intelligence among health professions' students in Jordan. BMC Med Inform Decis Mak.

[15] Alzaben AS, Almansour M, Alzahrani HS, Alrumaihi NA, Alhamedi NM, Albuhayjan NA, Aljammaz SA (2024). Development of valid and reliable questionnaire to evaluate knowledge, attitude, and practices (KAP) of lifestyle medicine domains. Healthcare (Basel).

[16] Trainor A, Richards JB (2021). Training medical educators to teach: bridging the gap between perception and reality. Isr J Health Policy Res.

[17] Patnaik S, Krishna S, Jain MK (2020). Knowledge, attitude, and practice regarding anaphylaxis among pediatric health care providers in a teaching hospital. J Child Sci.

[18] Zhang YB, He L, Gou L (2021). Knowledge, attitude, and practice of nurses in intensive care unit on preventing medical device-related pressure injury: a cross-sectional study in western China. Int Wound J.

[19] Kumah EA, McSherry R, Bettany-Saltikov J, van Schaik P, Hamilton S, Hogg J, Whittaker V (2022). Evidence-informed practice versus evidence-based practice educational interventions for improving knowledge, attitudes, understanding, and behavior towards the application of evidence into practice: a comprehensive systematic review of undergraduate students. Campbell Syst Rev.

[20] Valentin G, Nielsen CV, Nielsen AM (2023). Bridging inequity gaps in healthcare systems while educating future healthcare professionals—the social health bridge-building programme. Int J Environ Res Public Health.

[21] Hasan HE, Jaber D, Al Tabbah S, Lawand N, Habib HA, Farahat NM (2024). Knowledge, attitude and practice among pharmacy students and faculty members towards artificial intelligence in pharmacy practice: a multinational cross-sectional study. PLoS One.

